# Efficacy of salvage stereotactic radiotherapy for recurrent glioma: impact of tumor morphology and method of target delineation on local control

**DOI:** 10.1002/cam4.154

**Published:** 2013-10-28

**Authors:** Kengo Ogura, Takashi Mizowaki, Yoshiki Arakawa, Katsuyuki Sakanaka, Susumu Miyamoto, Masahiro Hiraoka

**Affiliations:** 1 Department of Radiation Oncology and Image-applied Therapy, Kyoto University Graduate School of Medicine54 Kawahara-cho Shogoin Sakyo-ku, Kyoto, 606-8507, Japan; 2 Department of Neurosurgery, Kyoto University Graduate School of Medicine54 Kawahara-cho Shogoin Sakyo-ku, Kyoto, 606-8507, Japan

**Keywords:** Local control, recurrent glioma, re-irradiation, salvage therapy, stereotactic radiotherapy, target delineation

## Abstract

In this study, we assessed the efficacy of salvage stereotactic radiotherapy (SRT) for recurrent glioma. From August 2008 to December 2012, 30 patients with recurrent glioma underwent salvage SRT. The initial histological diagnoses were World Health Organization (WHO) grades II, III, and IV in 6, 9, and 15 patients, respectively. Morphologically, the type of recurrence was classified as diffuse or other. Two methods of clinical target delineation were used: A, a contrast-enhancing tumor; or B, a contrast-enhancing tumor with a 3–10-mm margin and/or surrounding fluid attenuation inversion recovery (FLAIR) high-intensity areas. The prescribed dose was 22.5–35 Gy delivered in five fractions at an isocenter using a dynamic conformal arc technique. The overall survival (OS) and local control probability (LCP) after SRT were calculated using the Kaplan–Meier method. A univariate analysis was used to test the effect of clinical variables on OS/LCP. The median follow-up period was 272 days after SRT. The OS and LCP were 83% and 56% at 6 months after SRT, respectively. Morphologically, the tumor type correlated significantly with both OS and LCP (*P* = 0.006 and <0.001, respectively). The method of target delineation also had a significant influence on LCP (*P* = 0.016). Grade 3 radiation necrosis was observed in two patients according to Common Terminology Criteria for Adverse Events, version 3. Salvage SRT was safe and effective for recurrent glioma, especially non-diffuse recurrences. Improved local control might be obtained by adding a margin to contrast-enhancing tumors or including increased FLAIR high-intensity areas.

## Introduction

The management of recurrent glioma is a challenging issue. In particular, recurrent glioblastoma has a dismal prognosis; the median survival time after progression was 6 months in a clinical trial [1]. Various kinds of chemotherapy and targeted therapy have been tested, but the optimal treatment strategy remains unclear [2, 3]. Radiotherapy is one option. Although re-irradiation may not be a curative approach [4], it could be an attractive option for controlling progressive lesions at areas unsuitable for surgery and those remaining despite repeated chemotherapy. Stereotactic radiotherapy (SRT) may be used to spare as much normal brain tissue as possible. To date, several retrospective or phase I/II studies have been published [5–18]. The overall survival (OS) after salvage re-irradiation is reportedly about 10 months, but assessments of the local control probability (LCP) are rare. Local control and its palliative effect seem to be important endpoints in terms of local treatment. In this retrospective study, we focused on the relationships between LCP and several clinical factors, especially the tumor's morphological type and target delineation.

## Methods

### Patient population

**Figure 1:**
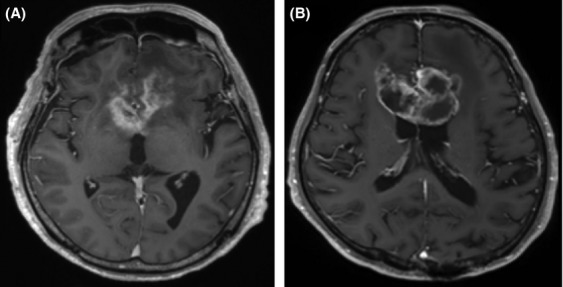
Examples of tumor morphological types. Representative cases of (A) diffuse and (B) non-diffuse recurrent tumors are shown. Diffuse recurrent tumors extended more than 2 cm from the primary site, with ≥50% of the margin qualitatively assessed as poorly defined.

From August of 2008 to December of 2012, 37 patients with recurrent glioma underwent salvage SRT at our hospital. Among them, seven patients who had disseminated disease at the time of SRT and/or had no follow-up magnetic resonance imaging (MRI) scans at least 1 month after initial SRT were excluded. The remaining 30 patients with 33 lesions were analyzed retrospectively, referring to clinical records. Written informed consent was obtained from the patients for publication of this report and any accompanying images. All patients underwent surgery and received radiotherapy with or without chemotherapy as initial treatments after the time of primary diagnosis. In our hospital, radiotherapy doses are 50.4–54 Gy in 28–30 fractions for low-grade glioma (WHO [World Health Organization] grade II) and 59.4–63 Gy in 30–35 fractions for high-grade glioma (WHO grade III–IV). Concurrent chemotherapy was combining nimustine (ACNU)–carboplatin–vincristine–interferon-*β* chemotherapy [19] for all grade gliomas, and after the emergence of temozolomide (TMZ) in our country, TMZ has been applied for high-grade gliomas. Two patients had received gamma-knife radiosurgery for low-grade glioma in other hospitals before our initial chemoradiotherapy treatment. Another patient had refused to continue initial radiotherapy and she had received only 18 Gy in 10 fractions. Regarding the morphological patterns of recurrent tumors on conventional MRI, Pope et al. [20, 21] classified them into four categories in the BRAIN trial: local, distant, diffuse, and multifocal. Using a modified version of this classification system, we classified the recurrent tumors into two groups: diffuse and other. Briefly, diffuse recurrence was defined as recurrence either centered or extending more than 2 cm (originally 3 cm) from the primary site or margin of the resection cavity, with ≥50% of the margin of the recurrent tumor qualitatively assessed as poorly defined. In contrast to a diffuse pattern, the margin of a recurrent tumor of another type (local, distant, or multifocal) was defined as mostly or completely well-defined. Details are given elsewhere [21]. Representative cases of diffuse and non-diffuse recurrent tumors are shown in Figure 1.

### SRT

Treatment was performed using the Novalis system, equipped with an ExacTrac system and Robotic Tilt Module mounted on the Exact Couch top (BrainLAB AG, Feldkirchen, Germany). Patients were immobilized in a thermoplastic stereotactic head mask with an additional bite block and infrared reflecting markers (BrainLAB AG). Patients were positioned using the Novalis/ExacTrac system, and positional errors, including translations and rotations, were corrected by moving the robotic couch.

For treatment planning, computed tomography (CT) scans (1.25 mm slice thickness) were acquired using a Light Speed RT scanner (GE Healthcare, Milwaukee, WI). Treatment planning was performed using iPlan or BrainScan software (BrainLAB AG). Images produced by conventional MRI were fused with the planning CT scans. The gross tumor volume (GTV) was defined as contrast-enhancing tumor. Delineation of the clinical target volume (CTV) was done at the discretion of the treating physician. We classified the groups retrospectively by two methods: A, contrast-enhancing tumor only (i.e., identical to the GTV); and B, contrast-enhancing tumor plus a margin of 3–10 mm and/or surrounding fluid attenuated inversion recovery (FLAIR) high-intensity increasing lesions. Then, the CTVs were expanded 1–2 mm to create the planning target volumes (PTVs) in consideration of setup error and patient motion. In one patient who had an absolutely non-contrast-enhancing tumor, the CTV was delineated based on a growing FLAIR high-intensity lesion (i.e., method B). The prescribed doses were specified at the isocenter; 22.5–35 Gy in five daily fractions was prescribed (median, 35 Gy). The PTV was covered by the 70–80% isodose line of the prescribed dose. In all patients, the dynamic conformal arc technique was used.

### Follow-up and assessment

We analyzed the intracranial status and disease progression after salvage SRT retrospectively, in accordance with “Response Assessment in Neuro-Oncology Working Group of the American Society of Clinical Oncology” (RANO) criteria [22]. Briefly, progression was defined as an increase in 25% of the product of perpendicular diameters of enhancing lesions, a significant increase in the T2/FLAIR non-enhancing component, appearance of new lesions, and clinical deterioration not attributable to causes other than the tumor or a reduction in the corticosteroid dose. The assessment of local control was also based on RANO criteria, considering contrast-enhancing lesions and/or T2/FLAIR components. Local recurrence patterns were operationally defined as “central” if the recurring tumors were centered within the initial contrast-enhancing tumor and otherwise as “marginal.” Treated lesions contrast-enhanced peripherally or heterogeneously, with no continuous progression, were diagnosed as radiation necrosis. Nuclear medicine tests, including positron emission tomography, were conducted as necessary for differential diagnosis. The severity of radiation necrosis was evaluated according to Common Terminology Criteria for Adverse Events (CTCAE), version 3, which defines asymptomatic central nervous system (CNS) necrosis with only radiographic findings as grade 1 toxicity. Grade 2 CNS necrosis is defined as symptomatic, but not interfering with the activities of daily living (ADL). Grade 3 CNS necrosis is symptomatic and interferes with ADL. Grade 4 CNS necrosis is defined as life-threatening and requires operative intervention. The level of steroid treatment and Eastern Cooperative Oncology Group performance status (PS) were also evaluated at the time of salvage SRT (baseline) and at 1 and 3 months after salvage SRT.

### Statistical analysis

OS, progression-free survival (PFS), and LCP were estimated using the Kaplan–Meier method. OS was calculated from the date of initial SRT to that of death or last follow-up. PFS was calculated from the date of initial SRT to that of disease progression, defined by RANO criteria, or last follow-up. LCP was calculated from the date of initial SRT to local failure or last imaging follow-up. A univariate analysis was used to estimate the association of OS or LCP with various clinical factors; *P*-values <0.05 were considered to indicate statistical significance. All analyses were performed with EZR (Saitama Medical Center, Jichi Medical University; http://www.jichi.ac.jp/saitama-sct/SaitamaHP.files/manual.html; Kanda, 2013), a graphical user interface for “R” software, version 2.13.0 (The R Foundation for Statistical Computing, Vienna, Austria). More precisely, it is a modified version of “R” Commander (version 1.6-3), designed to add statistical functions frequently used in biostatistics [23].

## Results

### Patient and tumor characteristics

The characteristics of 30 patients with 33 lesions are shown in Table 1. All patients received radiotherapy with or without chemotherapy at the time of primary diagnosis. In total, 24 recurrent tumors were within the initial radiotherapy field and the remaining nine were outside the field. The median time from initial radiotherapy to salvage SRT was 755 (range, 127–3571) days. SRT was performed for first recurrent tumors in nine (30%) patients and for repeated recurrent tumors in the remaining 21 (70%), who had already undergone salvage chemotherapy (20 patients) and/or salvage surgery (14 patient) more than 1 month before SRT. At the time of SRT, six patients underwent partial resections or biopsies with a histological diagnosis of recurrence while the remaining 24 patients were diagnosed radiographically or clinically because of difficulty in approaching the tumor location surgically or a poor PS. All recurrent tumors except one had a progressive contrast-enhancing component and were diagnosed clinically as WHO grade III or higher. One patient was initially diagnosed with anaplastic astrocytoma (WHO grade III). The median follow-up time from the start of SRT to death or last follow-up was 273.5 (range, 61–702) days, while the median MRI imaging follow-up period from SRT was 238 (range, 47–699) days.

**Table 1:** Characteristics of 30 patients with 33 lesions.

Characteristics	Number or value
Gender	
Male/female	17/13
Age (years)	
Median (range)	52.5 (19–81)
Primary diagnosis	
WHO grade II/III/IV	6/9/15
Most recent histological diagnosis	
WHO grade II/III/IV	2/10/18
Performance status	
0/1/2/3/4	9/10/5/4/2
Tumor location	
F/P/T/O/CC/BG/CB	9/1/5/3/6/5/4
Tumor morphological type	
Diffuse/other	11/22
Contrast-enhancing tumor volume (cc)	
Median (range)	3.2 (0–36.1)
PTV volume (cc)	
Median (range)	9.0 (1.0–140.0)
Target delineation	
Method A/B	16/17
Dose per fraction (Gy)	
4.5/5/6/7	1/3/8/21
Concurrent chemotherapy with SRT	
None/TMZ/ICE/Others	17/4/4/5

BG, basal ganglion; CB, cerebellum; CC, corpus callosum; F, frontal lobe; ICE, ifosfamide, carboplatin, and etoposide; O, occipital lobe; P, parietal lobe; SRT stereotactic radiotherapy; T, temporal lobe; TMZ, temozolomide; WHO, World Health Organization.

### Outcomes of SRT and influencing factors

**Figure 2:**
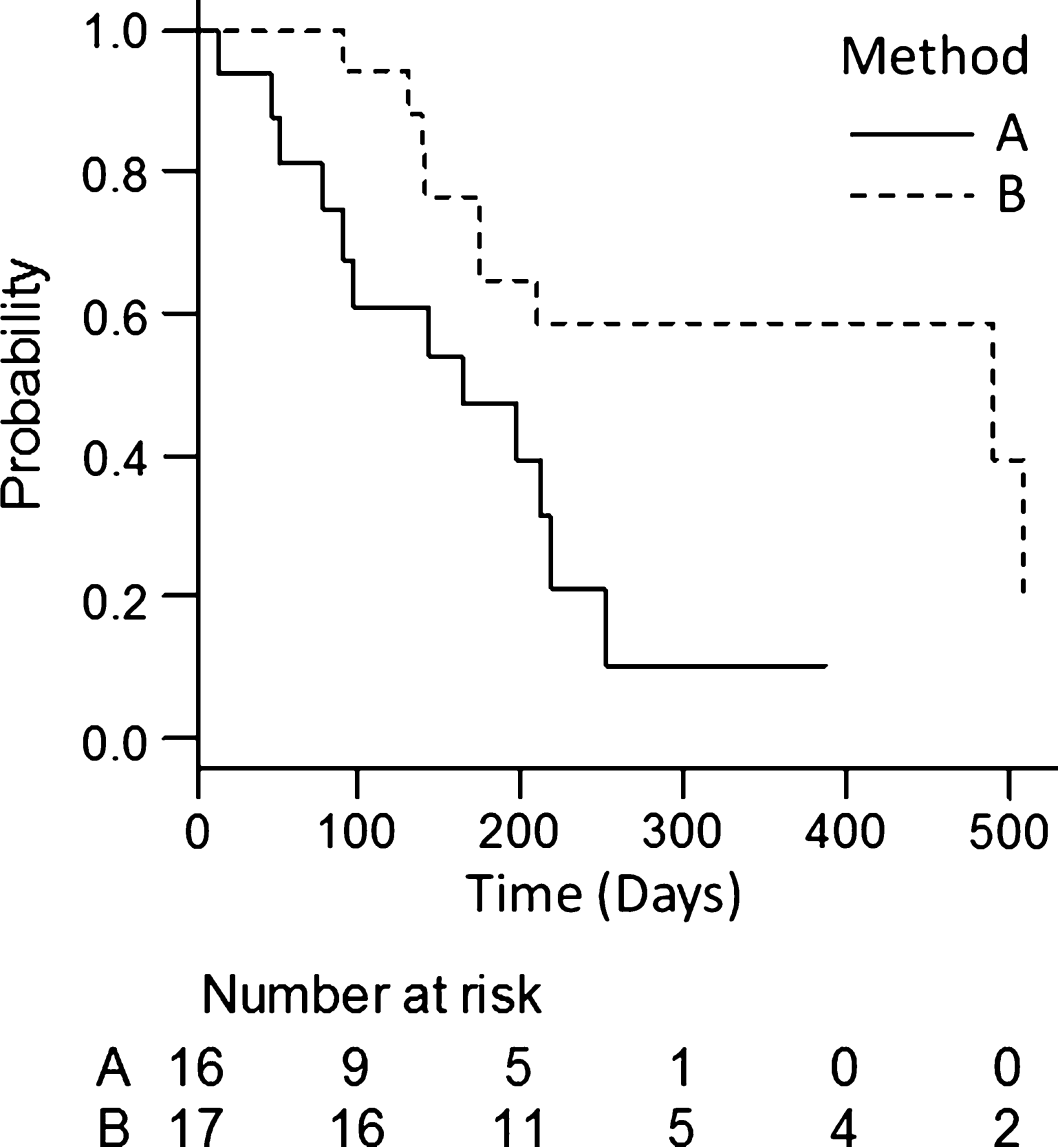
Local control probability depending on methods of target delineation. Local control probability of 33 lesions from the date of salvage stereotactic radiotherapy was estimated using the Kaplan–Meier method depending on methods of clinical target delineation: A, contrast-enhancing tumor only; or B, contrast-enhancing tumor plus a margin of 3–10 mm and/or surrounding fluid attenuated inversion recovery high-intensity increasing lesions.

The median OS was 316 days (95% confidence interval [CI], 252–389); at 6 and 12 months, the OS was 83% (95% CI, 64–93) and 34% (95% CI, 17–53), respectively. The median PFS was 91 days (95% CI, 75–121); at 6 and 12 months, the PFS was 19% (95% CI, 7.6–35) and 10% (95% CI, 2.2–26), respectively. The median LCP was 210 days (95% CI, 141–491); at 6 and 12 months, the LCP was 56% (95% CI, 37–71) and 38% (95% CI, 21–55), respectively. Local control failures were observed in 22 of 33 lesions (67%) within the follow-up time. The local failure patterns were central in eight (36%) and marginal in 14 (64%) lesions. Marginal recurrence after salvage SRT was observed in eight of 13 patients (62%) with method A and six of nine patients (67%) with method B. Kaplan–Meier curves of LCP for methods A and B are shown in Figure 2. Peripheral dose (minimum dose) of contrast-enhancing tumor was a median of 29.7 Gy (range, 17.8–31.3) in method A and 29.1 Gy (range, 18.8–32.2) in method B with no significant difference (Mann–Whitney *U* test; *P* = 0.79). A univariate analysis for OS and LCP was performed considering various clinical factors believed to be important; the results are summarized in Table 2. Morphological classification (diffuse or not), most recent WHO grade (II–III or IV), and PS were significantly associated with OS. On the other hand, morphological classification, PS, method of target delineation (contrast-enhancing tumor only or not), and the contrast-enhancing tumor volume (<4 cc vs. ≥4 cc) were significantly correlated with the LCP.

**Table 2:** Univariate analysis of overall survival and local control probability.

	Outcomes at 6months (95% CI) and *P*-values			
	
Factors	Overall survival		Local control	
Age (years)				
<50	85% (53–94)	0.69	60% (29–81)	0.60
≥50	82% (51–96)		53% (29–72)	
Performance status				
0–1	95% (68–99)	0.026^1^	76% (52–89)	<0.001^1^
2–4	61% (27–84)		14% (1–43)	
Most recent histological diagnosis				
WHO grade II–III	92% (54–99)	0.016^1^	64% (30–85)	0.21
WHO grade IV	78% (51–91)		52% (29–71)	
Time from initial RT to progression				
<600days	80% (51–93)	0.21	53% (29–72)	0.25
≥600days	86% (54–96)		61% (30–82)	
Concurrent chemotherapy				
Yes	68% (36–87)	0.20	38% (13–63)	0.24
No	94% (65–99)		68% (43–84)	
Tumor morphological type				
Diffuse	62% (28–84)	0.006^1^	21% (0.3–48)	<0.001^1^
Others	95% (68–99)		72% (49–87)	
Contrast-enhancing tumor volume (cc)				
<4cc	81% (51–93)	0.85	73% (46–88)	0.018^1^
≥4cc	86% (54–96)		33% (11–58)	
Target delineation				
Method A	73% (43–89)	0.084	47% (22–69)	0.016^1^
Method B	93% (61–99)		65% (38–82)	

CI, confidence interval; RT, radiotherapy; WHO, World Health Organization.

1 Regarded as statistically significant (*P*<0.05).

### Toxicity

Radiation necrosis was grade 1 in 13, grade 2 in five, and grade 3 in two lesions, according to CTCAE, version 3. No case of grade 4 toxicity was observed. Thus, the crude proportion of radiation necrosis ≥grade 3 was 6.1% (2/33 lesions) and no uncontrollable radiation necrosis was observed in the follow-up period. Fifteen patients received bevacizumab (BEV) after SRT and one before SRT. The median time from SRT to BEV was 148 (range, −15 to 514) days. One patient received BEV for the treatment of grade 3 radiation necrosis and the others did so as salvage treatment for progressive recurrent disease. A median PTV size was 7.2 cc (range, 1.4–47.8) in method A and 9.3 cc (range, 1.0–140.0) in method B with no significant difference (Mann–Whitney *U* test; *P* = 0.40). Radiation necrosis in method A was grade 1 in four patients, grade 2 in five patients and grade 3 in one patient. On the other hand, radiation necrosis in method B was grade 1 in nine patients and grade 2 in no patient, and grade 3 in one patient. In addition, there was no difference in usage of BEV between two groups (9/16 lesions in method A and 9/17 lesions in method B).

The steroid dose 1 month after SRT was decreased in three (10%), increased in three (10%), unchanged in five (17%), and was none in 19 (63%) patients. The reasons for increasing the steroid dose were a symptomatic edematous change surrounding the SRT-treated lesion in two patients and progressive disease in one. At 3 months after SRT, two patients had died of progressive disease: diffuse-invasive and infiltrative progression in one patient and disseminated progression in the other. The steroid dose was decreased in five and increased in five patients compared with the baseline dose. The remaining 18 patients needed no steroid treatment. The reasons for the increased steroid dose were progressive disease in three patients and a possible radiation-induced edematous change in two patients.

The PS 1 month after SRT was improved in four (13%), worsened in five (17%), and unchanged in 21 (70%) patients. The reasons for a decreased PS score were local or intracranial progression in four patients and acute radiation effects in one patient, as mentioned above. The PS score at 3 months after SRT was available in 28 patients (two had died, as mentioned above). Patients with an improved PS at 1 month after SRT remained so. An additional three patients had a worsened PS because of progressive disease in two patients and a complex partial seizure in one. The seizure was not the first, but the focus was considered to be the treated lesion and could have been due to an acute irradiation effect.

## Discussion

In this report, the presence of a diffuse-type tumor had significant effects on both OS and local control after salvage SRT. The definition of diffuse recurrence was based on Pope's classification in the BRAIN trial [20]. Although that system was created specifically for patients with recurrent glioblastoma who received BEV, it is useful for classifying various and complex morphological characteristics before and after salvage treatment. In some reports [24–26], diffuse-type recurrence has been discussed as a negative effect of antiangiogenic therapy. On the other hand, Wick et al. [27, 28] reported that diffuse-invasive recurrence (i.e., “gliomatosis-like phenotype”) might be a feature of late-stage glioma rather than a specific property of antiangiogenic treatment. In this study, we did not use antiangiogenic therapy at the time of first SRT except in one patient, and the poor outcomes observed in patients with a diffuse recurrence supports their finding. In our clinical course, about half of the patients (16 out of 30) received BEV treatment, but the OS was not significantly different from that of patients who did not receive BEV (*P* = 0.9 by univariate analysis).

In a univariate analysis, PS was also one of significant influencing factors on local control but it may be difficult to understand intuitively. In post hoc analysis, morphological type (diffuse or not) and PS (0–1 or 2–4) were strongly correlated with each other (Fisher's exact test; *P* = 0.001) and then an apparent influence of PS on LCP should be a reflection of that of morphological type on LCP. Diffuse-invasive recurrence and lower PS might also be two sides to a feature of late-stage recurrent glioma.

Delineation of the CTV has been limited to contrast-enhancing lesions in most recent reports [5, 9, 11, 14, 18]. In the re-irradiation setting, a smaller irradiated volume is obviously preferable in terms of toxicity, while limiting treatment to contrast-enhancing lesions might lead to a lower LCP, considering the invasiveness of gliomas. Especially in the case of diffuse tumors, a precise understanding of tumor spread is often difficult and supposedly amenable to local failure. In our analysis, the method of target delineation had an impact on LCP. There was no significant difference in background (PS, tumor morphological type, and contrast-enhancing volume) between the two methods of target delineation (Fisher's exact test; *P* > 0.1). There are few reports of target delineation using conventional MRI in the setting of salvage radiotherapy for recurrent glioma. Koga et al. [29] reported that extended-field stereotactic radiosurgery (SRS) yielded better local control for recurrent glioblastoma. They attached a 0.5- to 1-cm margin to contrast-enhancing lesions, and the toxicity profiles were reportedly tolerable, while the proportion of radiation necrosis in extended-field SRS seemed to be higher than with conventional SRS, approaching a significant level. Patel et al. [10] assessed 10 patients with recurrent glioblastoma who underwent SRT, and the re-irradiation volume was defined as a contrast-enhancing tumor with a rapid increase in the FLAIR imaging signal. The median PTV was 51.1 cc and the prescribed dose was 36 Gy in six fractions, twice weekly, with 90% coverage of the PTV. They reported that patients tolerated the treatment well with limited toxicity, while one patient underwent a biopsy and mixed residual tumor and necrosis was seen 11 months after SRT. Hundsberger et al. [17] reported that adding small margins to the gross target volume were counterintuitive and less appropriate. They attached a 2.5-cm margin to contrast-enhancing tumors and the surrounding edema. Despite this very large re-irradiation field, radiation necrosis was not observed in 10 patients treated with BEV while one patient of four that did not receive BEV showed radiation necrosis. Considering these findings, re-irradiation with an extended field and based on the premise of BEV treatment may be an attractive option for effective and safe salvage treatment.

**Figure 3:**
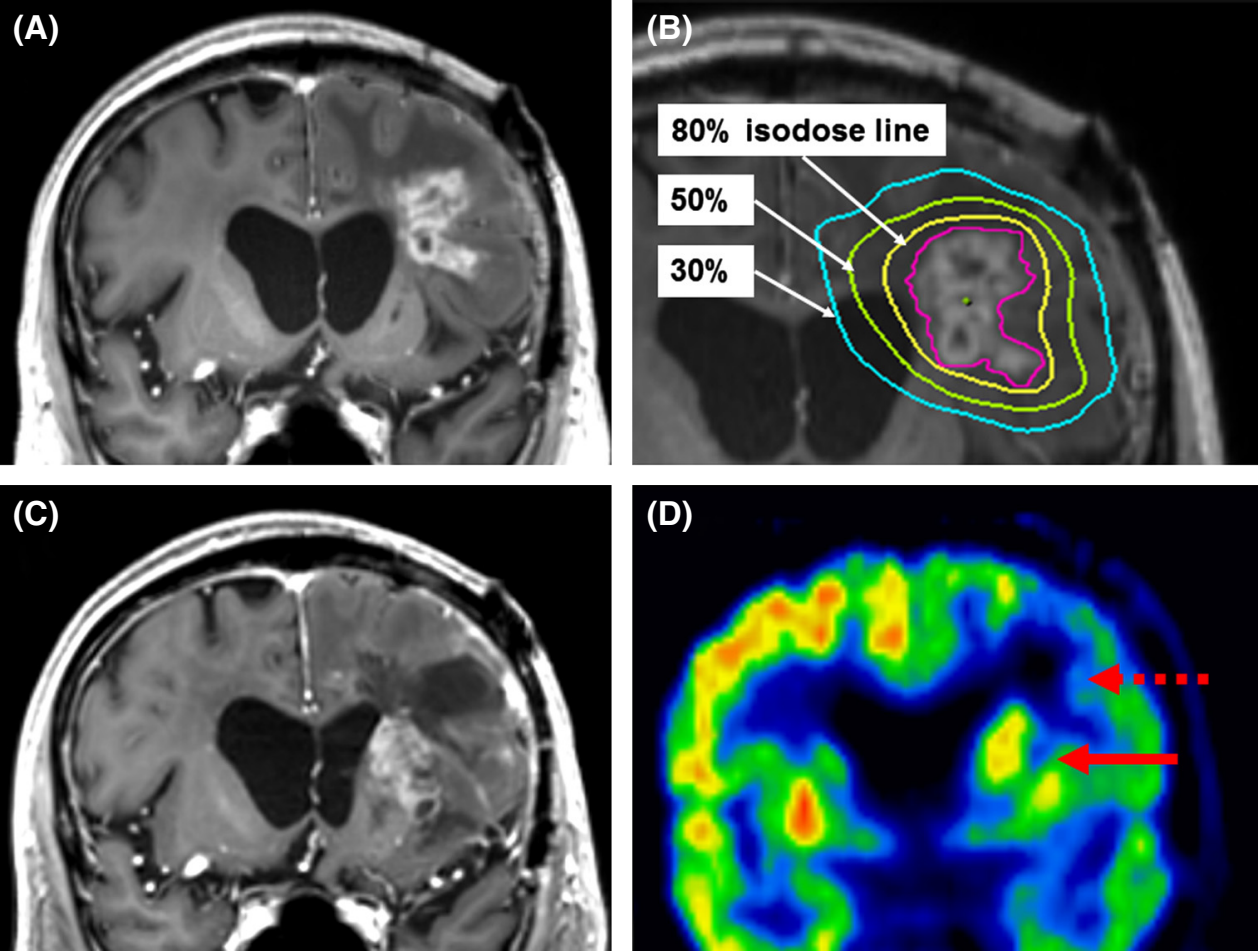
Representative case of marginal recurrence. (A) A recurrent tumor from an anaplastic oligoastrocytoma located in the left frontal lobe, adjacent to the initial surgical cavity and within the field of the initial radiotherapy. (B) The tumor was treated with salvage stereotactic radiotherapy (SRT). Target delineation of the planning target volume (PTV, indicated by the magenta line) was classified as method A (contrast-enhancing lesion plus a 1-mm margin); the prescribed dose was 35 Gy in five fractions with 80% coverage of the PTV. (C) At 10 months after treatment, a new recurrent tumor at the left basal ganglion emerged, adjacent to the previously treated lesion (marginal recurrence). (D) L-Methyl-^11^C-methionine positron emission tomography supported the diagnosis of recurrence (indicated by a solid arrow), while the SRT-treated area was determined to be radiation necrosis (indicated by a dashed arrow).

In our series, the proportion of marginal recurrence was higher than that of central recurrence. A representative case of marginal recurrence is shown in Figure 3. In such a case, adding margins to the contrast-enhancing tumor or target delineation based on successive FLAIR imaging might yield better local control. Although the risk of radiation necrosis might be increased, the incidence of radiation necrosis in our series was not different between methods A and B. On the other hand, marginal recurrence after salvage SRT was similarly observed between methods A and B; thus, method B might yield extended control over tumor recurrence compared to method A, but it did not change the local failure pattern after salvage SRT. Additionally, whole intracranial control (PFS) was very poor (the 6-month PFS was only 19%). This was a reflection of other new multifocal, subependymal, or disseminated recurrences after SRT. Most patients (21/30; 70%) suffered from resistant recurrent tumors despite repeated salvage treatment before SRT. These dismal patterns of recurrence may also be a feature of late-stage glioma; therefore, not only improved local therapy but also effective systemic therapy should be considered.

Radiation necrosis is a major concern in the re-irradiation setting. In this report, two lesions in two patients had CTCAE grade 3 necrosis. Both were within the initial radiotherapy field (63 Gy in 35 fractions and 60 Gy in 30 fractions) and treated at a dose of 35 Gy in five fractions with methods A and B for target delineation. The PTVs were 6.8 and 3.0 cc, respectively. The crude proportion of grade 3 necrosis (6.1%) here was comparable with that in recent reports. The dose per fraction in our report was higher than that in other recent series, whereas the PTV was smaller. Our prescribed dose might have a higher risk when applied to much larger tumors. Ernst-Stecken et al. [5] reported outcomes for nearly the same dose-fraction schedule (35 Gy in five fractions with 90% covering the PTV) and for larger volumes (a median PTV of 22.4 cc) and showed acceptable toxicity and maintenance of quality of life (QOL). They also reported that the PTVs were larger than 60 cc in all patients with increased edema after 3 months, with no apparent progression. Considering the difficulty of intracranial control, salvage SRT alone is not a curative approach and the balance between better local control and acceptable toxicity is important. The palliative effect and QOL after SRT should be evaluated in a prospective manner.

As is typical, this retrospective study has some limitations. The small sample size, selection bias, lack of biological information, and various treatment factors, including chemotherapy before and after SRT, made it difficult to interpret the patient outcomes. However, there are few data about salvage SRT for recurrent glioma and the sample size in our study was similar to those in the literature. In particular, our analysis provides additional data about LCP in terms of tumor morphology and method of target delineation.

In conclusion, salvage SRT for recurrent glioma was safe and yielded better outcomes in patients with non-diffuse recurrent tumors. Improved local control may be obtained by adding a margin to contrast-enhancing tumors or including increased FLAIR high-intensity areas, while the overall intracranial control was very poor. Thus, there is continuing need for systemic therapy or a new modality to prevent remote recurrences.
